# Rabbit PrP Is Partially Resistant to *in vitro* Aggregation Induced by Different Biological Cofactors

**DOI:** 10.3389/fnins.2021.689315

**Published:** 2021-06-18

**Authors:** Juliana N. Angelli, Yulli M. Passos, Julyana M. A. Brito, Jerson L. Silva, Yraima Cordeiro, Tuane C. R. G. Vieira

**Affiliations:** ^1^Federal Institute of Rio de Janeiro, Rio de Janeiro, Brazil; ^2^Institute of Medical Biochemistry Leopoldo de Meis, National Institute of Science and Technology for Structural Biology and Bioimaging, Federal University of Rio de Janeiro, Rio de Janeiro, Brazil; ^3^Department of Pharmaceutical Biotechnology, Faculty of Pharmacy, Federal University of Rio de Janeiro, Rio de Janeiro, Brazil

**Keywords:** prion, rabbit PrP, resistance, conversion, cofactor, DNA, glycosaminoglycan, lipid

## Abstract

Prion diseases have been described in humans and other mammals, including sheep, goats, cattle, and deer. Since mice, hamsters, and cats are susceptible to prion infection, they are often used to study the mechanisms of prion infection and conversion. Mammals, such as horses and dogs, however, do not naturally contract the disease and are resistant to infection, while others, like rabbits, have exhibited low susceptibility. Infection involves the conversion of the cellular prion protein (PrP^C^) to the scrapie form (PrP^Sc^), and several cofactors have already been identified as important adjuvants in this process, such as glycosaminoglycans (GAGs), lipids, and nucleic acids. The molecular mechanisms that determine transmissibility between species remain unclear, as well as the barriers to transmission. In this study, we examine the interaction of recombinant rabbit PrP^C^ (RaPrP) with different biological cofactors such as GAGs (heparin and dermatan sulfate), phosphatidic acid, and DNA oligonucleotides (A1 and D67) to evaluate the importance of these cofactors in modulating the aggregation of rabbit PrP and explain the animal’s different degrees of resistance to infection. We used spectroscopic and chromatographic approaches to evaluate the interaction with cofactors and their effect on RaPrP aggregation, which we compared with murine PrP (MuPrP). Our data show that all cofactors induce RaPrP aggregation and exhibit pH dependence. However, RaPrP aggregated to a lesser extent than MuPrP in the presence of any of the cofactors tested. The binding affinity with cofactors does not correlate with these low levels of aggregation, suggesting that the latter are related to the stability of PrP at acidic pH. The absence of the N-terminus affected the interaction with cofactors, influencing the efficiency of aggregation. These findings demonstrate that the interaction with polyanionic cofactors is related to rabbit PrP being less susceptible to aggregation *in vitro* and that the N-terminal domain is important to the efficiency of conversion, increasing the interaction with cofactors. The decreased effect of cofactors in rabbit PrP likely explains its lower propensity to prion conversion.

## Introduction

The cellular prion protein (PrP^C^) is a constitutive protein that is mostly found attached to the extracellular membrane. PrP^C^ occurs naturally in the cells of all mammals, primarily in the nervous system (Prusiner, 1991), and is a conserved protein that exhibits a high sequence and structural identity. The protein has an N-terminal unstructured and flexible region formed by residues 23 to 121. It has an octapeptide region known as an octarepeat, which is comprised of a sequence of eight amino acids (PHGGGWGQ) that are repeated five times. The globular C-terminal region is composed of residues 122 to 231, which have three α-helices (H1 to H3), with H2 and H3 connected by a disulfide bridge and one small antiparallel β-sheet (Hornemann et al., 1997). Minor differences have been observed between the globular domain conformation in different species related to specific amino acid substitutions, including surface charge potential and dynamics (Pastore and Zagari, 2007; Wen et al., 2010a; Srivastava and Lapidus, 2017).

The prion protein has been physiologically related to several functions in the central nervous system, including neuronal protection, neuroplasticity, and cell signaling (Chen et al., 2003; Lopes et al., 2005; Linden, 2017; Wulf et al., 2017). However, it is most prominently associated with the development of pathologies known as transmissible spongiform encephalopathies (TSEs) or prion diseases. PrP^C^ can undergo changes in its native conformation that turn it into its pathogenic isoform, PrP scrapie (PrP^Sc^), a structure rich in β-sheets (Prusiner, 1982). PrP^Sc^ can arise because of errors in the protein folding process, post-translational conformational changes, and conversion by direct contact between PrP^Sc^ and PrP^C^ in an autocatalytic process (Castilla and Requena, 2015).

Naturally occurring TSEs have been described in several mammal species. These include scrapie in sheep and goats, bovine spongiform encephalopathy (BSE) in cattle, chronic wasting disease (CWD) in deer, and Creutzfeldt-Jakob disease (CJD) in humans. Scrapie has been experimentally transmitted to rats and cats, although with varying degrees of difficulty (Gibbs and Gajdusek, 1973). The molecular mechanisms that determine transmissibility between species and barriers to transmission remain poorly understood (Sweeting et al., 2010). For example, some species do not develop TSEs at all, such as rabbits, dogs, and horses (Sigurdson and Miller, 2003; Bian et al., 2017). These mammals’ PrP^C^ have been reported as being resistant to conversion by PrP^Sc^ samples from other species (Vorberg et al., 2003; Polymenidou et al., 2008). However, prions have been generated *in vitro* from rabbit material using protein misfolding cyclic amplification (PMCA), and are capable of infecting leporids (Chianini et al., 2012) and transgenic mice expressing rabbit PrP (Vidal et al., 2015). Species previously considered completely resistant to prions have therefore been shown to be slightly susceptible to TSE infections (Castilla et al., 2005; Saá et al., 2006; Chianini et al., 2013; Vidal et al., 2015).

**FIGURE 1:**
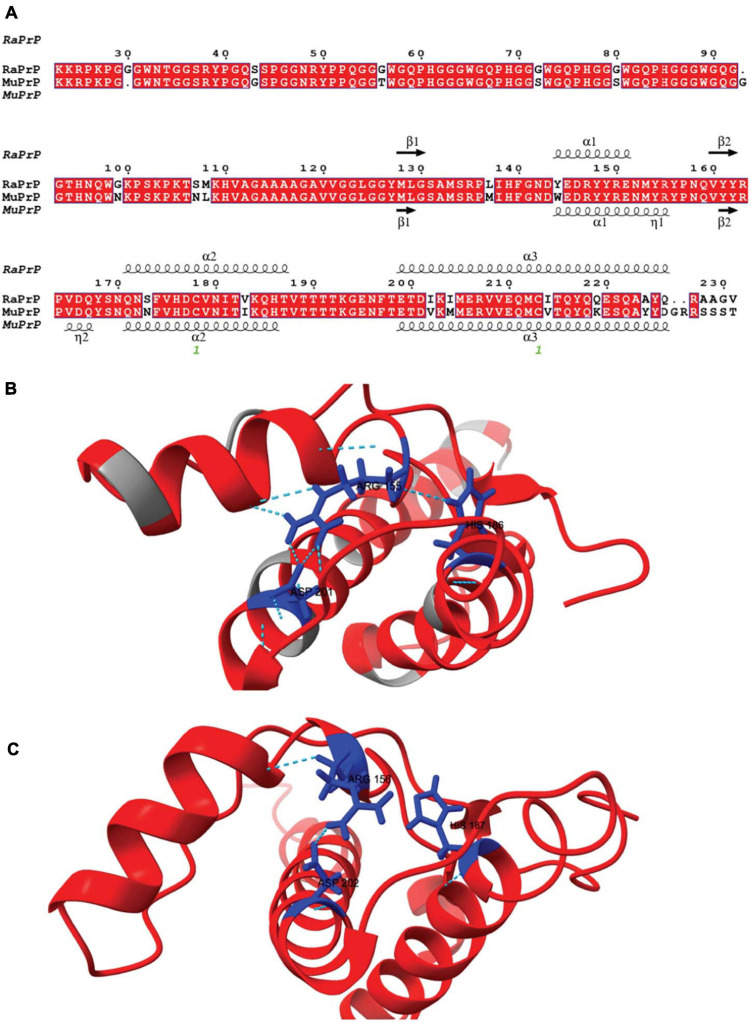
Rabbit and murine PrP structural alignment and comparison. **(A)** RaPrP^23–231^ sequence (UNIPROT Q95211) was aligned with MuPrP^23–231^ sequence (UNIPROT P04925) using the T-Coffee server. The figure was produced with the ESPrip server, using PDB 2FJ3 for RaPrP^23–231^ and PDB 1XYX for MuPrP^23–231^ secondary structure depiction. Aligned amino acid residues are in red boxes to distinguish them from low similarity residues. The secondary structures (alpha-helix and beta-strands) are shown on the top of the alignment for RaPrP^23–231^ and the bottom for MuPrP^23–231^. **(B)** RaPrP^23–231^ 3D structure is shown in red, and non-homologous residues are colored gray. ARG 155, HIS186, and ASP201 (rabbit sequence numbering) are depicted in blue, showing hydrogen bonds and saline bridges between these residues. **(C)** MuPrP^23–231^ 3D structure in red with ARG 156, HIS187, and ASP 202 (mouse sequence numbering) are depicted in blue. No hydrogen bond is observed between ARG 156 and HIS187, and ARG 156-ASP 202 shows one salt bridge. ChimeraX was used for structure visualization and H-bond and salt bridge identification.

Although human and rabbit PrPs have very similar primary sequences (88%) (Yan et al., 2014), some amino acid residues in the rabbit PrP^C^ sequence appear to contribute significantly to its inability to convert into PrP^Sc^ and are thus closely associated with rabbits’ low susceptibility to TSEs (Loftus and Rogers, 1997; Vorberg et al., 2003; Wen et al., 2010b; Yuan et al., 2013; Eraña et al., 2017). The primary sequence alignment between rabbit and mouse PrP has nine different amino acid residues in the flexible N-terminus and 14 in the structured C-terminus (Figure 1). In rabbits, PrP has a larger positively charged surface than it does in mice (Wen et al., 2010a; Silva et al., 2011).

Although the conversion of PrP^C^ is caused by PrP^Sc^, several cofactors have been identified as important adjuvants in this process. These include glycosaminoglycans (GAGs), lipids, and nucleic acids (Deleault et al., 2007; Silva and Cordeiro, 2016; Vieira and Silva, 2016).

Several studies have linked GAGs to prion biology. Dermatan sulfate is one of the most commonly found components in amyloid deposits (Hirschfield and Hawkins, 2003). Membrane heparan sulfate plays a crucial role in the development of prion disease as a receptor for PrP^Sc^ (Horonchik et al., 2005; Vieira and Silva, 2016). However, while these molecules have been observed to stimulate conversion in some instances, at other times, they inhibit the conversion, thus showing a paradoxical effect. Low molecular weight heparin interacts with murine PrP, leading to a transient oligomerization/aggregation process (Vieira et al., 2011). The octapeptide region has also been shown to be important for interaction at neutral pH (Vieira et al., 2011; Kim et al., 2020), while a second binding site in the C-terminal region of the protein under acidic conditions has also been suggested (Vieira et al., 2011). In addition, low molecular weight heparin acts as an inhibitor of PrP fibrillation, indicating its therapeutic potential (Vieira et al., 2011, 2014).

Lipids also play a role in the conversion of PrP^C^, and some phospholipids have been described as important for conversion and aggregation (Supattapone, 2014). Lipids such as phosphatidylserine (PS), phosphatidic acid (PA), phosphatidylethanolamine (PE), and phosphatidylinositol (PI) lead to the aggregation of PrP^C^ (Tsiroulnikov et al., 2009), converting it to a protease-resistant form (Wang et al., 2007). Palmitoyloleoyl-phosphatidylglycerol (POPG) vesicles have been shown to induce PrP structural changes, leading to intermediates that, with the addition of RNA, form aggregates with infectious characteristics (Miller et al., 2013).

Besides lipids, GAGs, and proteins (Linden et al., 2008; Satoh et al., 2009), PrP also binds nucleic acids, both DNA and RNA. These interactions have been the subject of research over recent decades. Nucleic acids have been proposed to act as catalysts in the conversion of PrP^C^ to a PrP^Sc^-like form (Cordeiro et al., 2001; Cordeiro and Silva, 2005). More recently, DNA-induced prion-like conversion in a physiological process was also observed, with specific DNA aptamers modulating PrP liquid-liquid phase separation (Matos et al., 2020). The binding of nucleic acids can thus influence the unfolding of proteins in both disease-related and physiological situations.

Some regions of murine PrP have already been observed to bind to nucleic acids, such as the lysine-rich regions 23–27 and 101–110 and the C-terminal globular domain (Lima et al., 2006; Cavaliere et al., 2013). DNA can bind to PrP *in vitro*, modulating protein aggregation; this interplay changes the native conformation of PrP and increases the presence of β-sheets (Cordeiro et al., 2001; Nandi et al., 2002). A complex formed by the interaction with a GC-rich 21 bp DNA (D67) has been shown to be toxic to a murine neuroblastoma cell line (Macedo et al., 2012). Recently, PrP-DNA interactions were investigated in a more targeted manner, selecting short single-stranded NA sequences that bind with high affinity and specificity to the extended C-terminal domain of the prion protein (PrP^90–231^). A selected 25-mer apatmer (A1) was able to modulate the phase transition of PrP – from LLPS to aggregation (Matos et al., 2020).

Since several cofactors are important in modulating the conversion and aggregation of PrP, the interaction of these molecules with different PrP sequences may help explain differences in susceptibility between species. In this study, we compare the interactions of rabbit and mouse PrP with different biological cofactors such as GAGs (heparin and dermatan sulfate), phosphatidic acid (PA), and DNA oligonucleotides (A1 and D67), demonstrating that the interaction with these cofactors is related to a lower susceptibility of RaPrP to aggregation *in vitro*.

## Materials and Methods

### Glycosaminoglycans

Dermatan sulfate from porcine intestinal mucosa (code C3788, M.W. 30 kDa avg.) was purchased from Sigma-Aldrich (St. Louis, MO, United States). Unfractionated heparin (code 2608411, M.W. 15 kDa avg.) was purchased from Merck (Darmstadt, Germany).

### Preparation of PA Large Unilamellar Vesicles

Phosphatidic acid (L-α-phosphatidic acid, monosodium salt of chicken egg, cod. 840101) was purchased from Avanti Polar Lipids, Inc. (Alabaster, AL, United States). Powdered PA was solubilized in chloroform (code 102445, Merck, Darmstadt, Germany). For the formation of the lipid film, chloroform was evaporated using nitrogen gas. Subsequently, the film was resuspended in PBS buffer. To form large unilamellar vesicles (LUVs), the sample was extruded (code 610000) against polycarbonate membranes with a pore diameter of 0.1 μm (code 610005), all from Avanti Polar Lipids, Inc. (Alabaster, AL, United States).

### DNA Oligonucleotides

Two DNA sequences previously studied by the group were chosen, one single-stranded (A1) and one double-stranded (D67). A1 contains 25 nucleotides (5′-CCGCGTACAATCGAGCTCGGGTGTC-3′) and D67 has 21 nucleotides (5′-AAAGGACGCGCGCGCGCGTTA-3′). The oligonucleotides were synthesized and purified by Integrated DNA Technologies (Coralville, IA, United States). The DNA material was annealed by heating at 95°C for 5 min and subsequently slow cooling overnight before the experiments. The DNA concentration was determined by absorbance at 260 nm using the corresponding molar extinction coefficient of each oligonucleotide.

### Prion Protein Expression and Purification

The expression in *E. coli* and purification by high-affinity chromatography of recombinant full length (PrP^23–231^) and truncated (PrP^90–231^) murine (Mu) and rabbit (Ra) proteins were performed as described in Vieira and Silva (2019).

### Spectroscopic Measurements

Protein intrinsic fluorescence and light scattering spectra were acquired using an FP-8200 spectrofluorometer (Jasco Corp., Tokyo, Japan) or a PC1 spectrofluorometer (ISS, Champaign, IL, United States) in an “L” geometry (at 90° relative to the excitation light). All samples were prepared in one of two solutions at 25°C: 50 mM Tris (pH 7.4) and 100 mM NaCl, or 20 mM sodium acetate buffer (pH 5.5), and 100 mM NaCl. For all PrP constructs, the concentration used was 2 μM for interactions with GAG and PA. To assess the interaction with the DNA sequences, a concentration of 5 μM was used for PrP^90–231^ constructs and 2 μM for PrP^23–231^ due to signal intensities. For light scattering, the samples were illuminated at 450 nm, and data were acquired from 430 to 470 nm (DNA analysis) or else at 320 nm with data acquisition from 300 to 340 nm (GAG and PA analysis). Intrinsic fluorescence measurements were performed by exciting the sample at 290 nm and collecting the fluorescence emissions between 300 and 420 nm (DNA analysis) or exciting the sample at 280 nm and collecting the fluorescence emissions between 300 and 420 nm. The Stern-Volmer constant (K_SV_) was obtained from the linear regression equation of fluorescence data (F_0_/F) from quencher cofactors and can be interpreted as the association constant of the complex (K_a_) according to the following equation (Jameson, 2014):

(1) F/0F=1+K[Q]a

Where F_0_ is the free protein initial fluorescence and F is the fluorescence signal at each quencher concentration, represented by [Q]. PrP interaction with PA data were analyzed as the center of spectral mass and fitted using GraphPad Prism with a one-site binding non-linear regression:

(2) ΔCM=Bmax[PA]/K+d[PA]

Where ΔCM is the variation of the center of fluorescence spectral mass, Bmax is the maximum number of binding sites, [PA] is the concentration of PA, and K_d_ is the equilibrium dissociation constant. K_d_ was converted to its inverse, K_a_.

PrP:GAG fluorescence was measured after protein disaggregation. Samples were prepared at each specified PrP:GAG concentration in Eppendorf Protein LoBind Tubes at 25°C, and measurements were taken after 4 h.

### Heparin Affinity Chromatography

A HiTrap Heparin column was used (code 17040701, GE Healthcare, Little Chalfont, United Kingdom). The PrP sample was applied to the column, followed by a washing step with 20 mL of buffer (20 mM sodium acetate solution at pH 5.5, or 50 mM Tris solution at pH 7.4). Protein elution was carried out with 70 mL of 20 mM sodium acetate with 1 M NaCl at pH 5.5, or 50 mM Tris solution with 1 M NaCl at pH 7.4. The flow rate used in all steps was 1 mL/min. The data were normalized by the maximum absorption at 280 nm of each tested condition, and negative absorbance values were considered equal to zero. Retention factor values were calculated to allow comparison of the relative affinities for the immobilized cofactor. It is calculated by the formula (Gjerde et al., 2009):

(3) k=(t-rt)m/tm

Where t_*r*_ is the retention time of the test substance, and t_m_ is the column dead time.

## Results

### Aggregation and Interaction of Murine and Rabbit PrP With GAGs

In the cell, GAGs (especially heparan sulfate) and PrP molecules can both be found in the plasma membrane and endocytic compartments (Godsave et al., 2015) and thus are able to experience pH values close to neutral and acid (Silva et al., 2011). We therefore evaluated the PrP:low molecular weight heparin interaction at these two pH values. We found an interaction with a binding site in the N-terminal domain of murine PrP (MuPrP) at pH 7.4 and an interaction with a second site located at the C-terminal domain of MuPrP at pH 5.5 (Vieira et al., 2011). Low molecular weight heparin was also shown to induce transient aggregation in MuPrP (Vieira et al., 2011).

**FIGURE 2:**
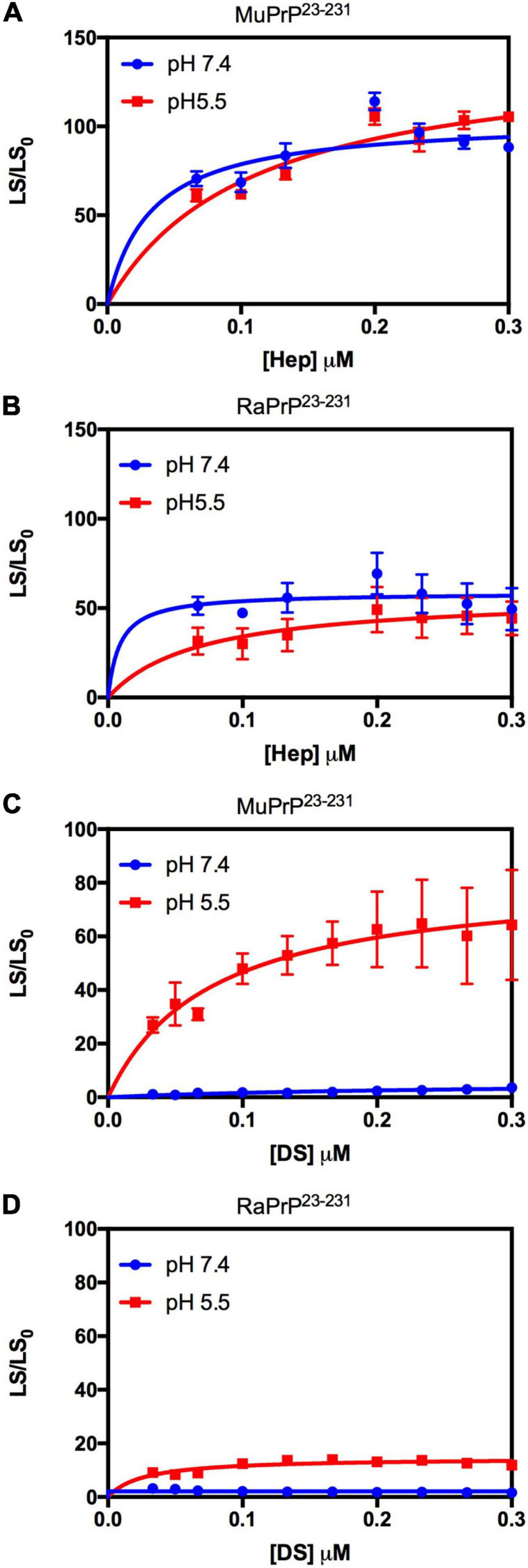
Rabbit PrP^23–231^ aggregates less in the presence of GAGs. Effect of increasing concentrations of Hep **(A,B)** and DS **(C,D)** on the aggregation of MuPrP^23–231^ (2 μM) **(A,C)** and RaPrP^23–231^ (2 μM) **(B,D)** at pH 5.5 (red) and 7.4 (blue). Representative data from three experiments. The experiments were performed in 50 mM Tris buffer containing 100 mM NaCl at pH 7.4 or in 20 mM sodium acetate buffer containing 100 mM NaCl at pH 5.5. All experiments were performed at 25°C. The error bars represent the standard deviation of at least two independent experiments.

We used light scattering measures to evaluate and compare the effect of GAGs on the aggregation of MuPrP^23–231^ and RaPrP^23–231^. The results showed that increasing heparin concentrations at pH 7.4 and 5.5 were accompanied by an increase in light scattering for MuPrP^23–231^ and RaPrP^23–231^, suggesting the formation of aggregates (Figures 2A,B, respectively). However, the effect was 200% greater in MuPrP^23–231^ (Figure 2A) than in RaPrP^23–231^ (Figure 2B). Meanwhile, dermatan sulfate triggered aggregation only at pH 5.5 in MuPrP^23–231^ and RaPrP^23–231^ (Figures 2C,D). The aggregation was 60% greater for MuPrP, further demonstrating the contrasting effect between the two PrPs at an acidic pH. Aggregation was transient in all conditions (Supplementary Figures 1A,B), consistent with observations for low molecular weight heparin with MuPrP (Vieira et al., 2011).

**FIGURE 3:**
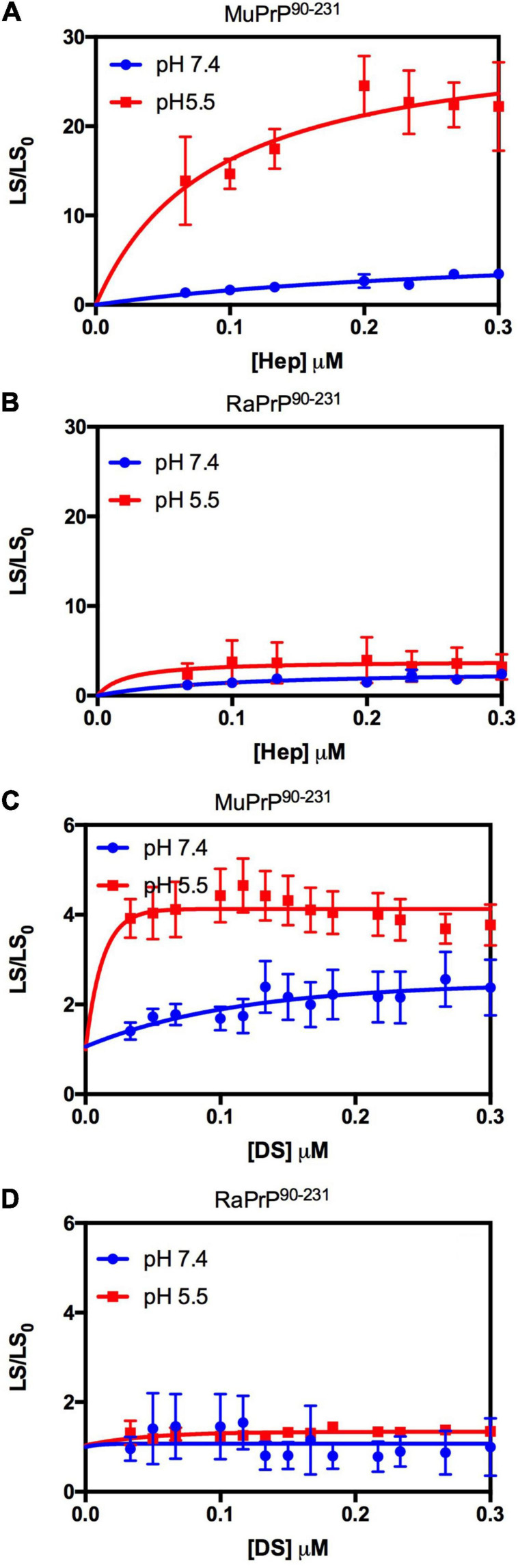
Rabbit PrP^90–231^ aggregates less in the presence of GAGs. Effect of increasing concentrations of Hep **(A,B)** and DS **(C,D)** on the aggregation of MuPrP^90–231^ (2 μM) **(A,C)** and RaPrP^90–231^ (2 μM) **(B,D)** at pH 5.5 (red) and 7.4 (blue). The experiments were performed in 50 mM Tris buffer containing 100 mM NaCl at pH 7.4 or in 20 mM sodium acetate buffer containing 100 mM NaCl at pH 5.5. All experiments were performed at 25°C. The error bars represent the standard deviation of at least two independent experiments.

We also compared the interaction of GAGs with PrP^90–231^ to explore the importance of the unstructured N-terminal region for the observed effect (Figure 3). A robust aggregation was only observed at pH 5.5 for heparin (Figure 3A), though the effect was 500% lesser than for PrP^23–231^, corroborating data obtained for low molecular weight heparin that showed only one binding site at the C-terminal domain at acidic pH (Vieira et al., 2011). The same effect was observed for dermatan sulfate (Figure 3C), suggesting that heparin and dermatan sulfate share the same binding regions but with different affinities. RaPrP^90–231^ showed a negligible aggregation for both heparin (Figure 3B) and dermatan sulfate (Figure 3D).

**FIGURE 4:**
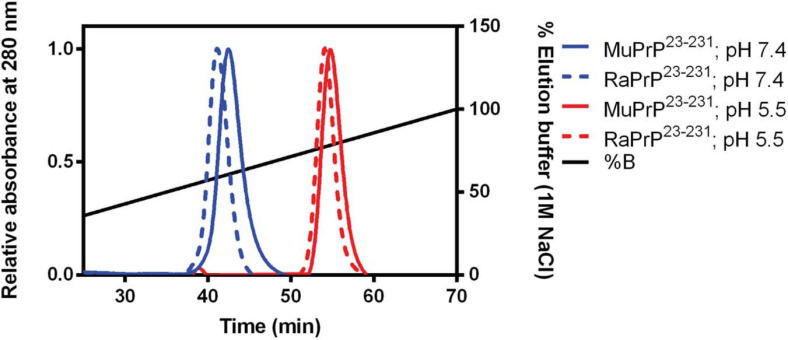
Murine and Rabbit PrP^23–231^ interaction with Heparin. Affinity chromatography of a heparin column of MuPrP^23–231^ (solid) and RaPrP^23–231^ (dotted) at pH 7.4 (in blue) and pH 5.5 (in red). Representative data from three experiments.

To evaluate binding and affinities, we performed affinity chromatography with a heparin column (Figure 4). The result showed that all PrP^23–231^ types were able to interact with the resin, with a higher concentration of NaCl required to displace this interaction at pH 5.5. A retention factor (k) difference between pH 5.5 and 7.4 was of 12.3 for MuPrP and 13 for RaPrP, reflecting a greater affinity at pH 5.5. We observed that k difference between MuPrP^23–231^ and RaPrP^23–231^ was of 1.4 at pH 7.4 and 0.7 at pH 5.5, suggesting a low difference in the affinities using this methodology (Figure 4).

**FIGURE 5:**
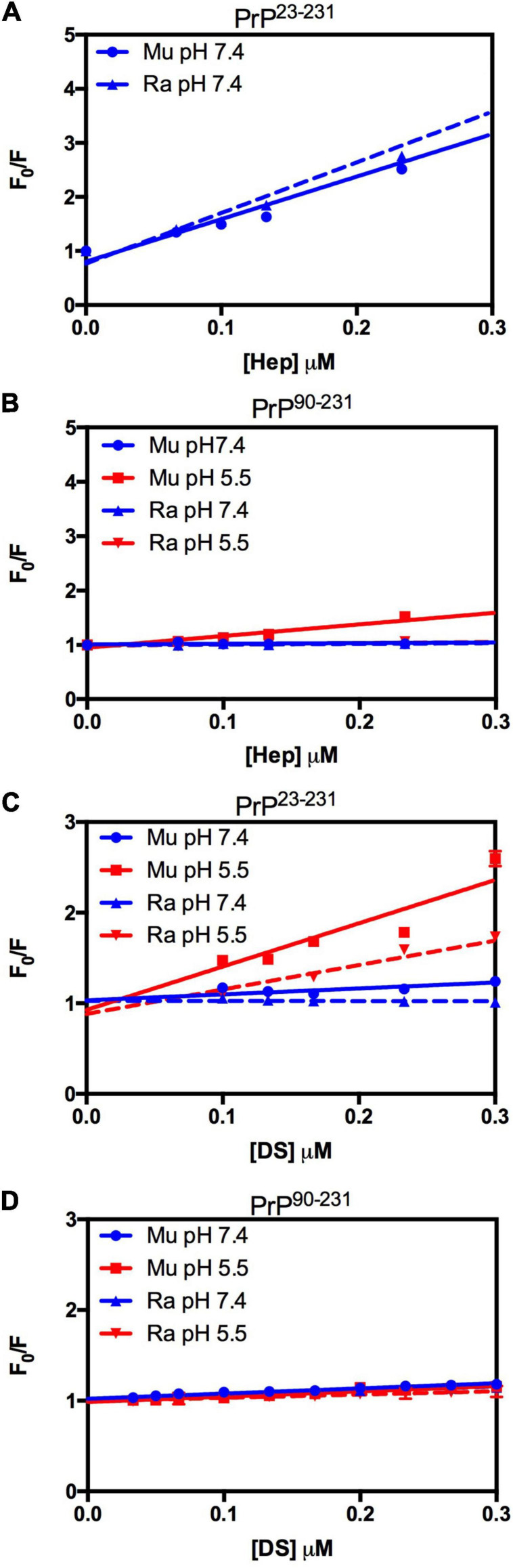
RaPrP^23–231^ interacts less with GAGs than MuPrP at pH 5.5, and the N-terminus is important for this interaction. Effect of increasing concentrations of Hep **(A,B)** and DS **(C,D)** on the fluorescence emission of PrP^23–231^ (2 μM) **(A,C)** and PrP^90–231^ (2 μM) **(B,D)** at pH 5.5 (red) and 7.4 (blue). Representative data from three experiments. The experiments were performed in 50 mM Tris buffer containing 100 mM NaCl at pH 7.4 or in 20 mM sodium acetate buffer containing 100 mM NaCl at pH 5.5. All experiments were performed at 25°C. The error bars represent the standard deviation of at least two independent experiments.

To further analyze affinity differences, we also performed protein intrinsic fluorescence measurements (Figure 5) after protein disaggregation. The results showed that binding led to a decrease in fluorescence intensity with increasing concentrations of heparin (Figures 5A,B) and dermatan sulfate (Figures 5C,D) at pH 7.4 and 5.5. Binding therefore led to fluorescence quenching, probably due to the approximation of glucosamine and galactosamine moieties (by heparin and dermatan sulfate, respectively) to tryptophan residues. Table 1 shows the results of the Stern-Volmer linear regression to compare affinity. All conditions were analyzed except for the interaction of PrP^23–231^ (murine and rabbit) with heparin at pH 5.5 since disaggregation was not complete under heparin treatment (Supplementary Figure 1C), and remaining aggregates can interfere with intensity measurements, promoting an inner filter effect (Jameson, 2014).

**TABLE 1:** Observed association constants for all cofactors studied.

**Sample**	**K_a_ (μM^–1^)**			
	**PrP^23–231^**		**PrP^90–231^**	
	**pH 5.5**	**pH 7.4**	**pH 5.5**	**pH 7.4**
MuPrP:Hep	NA	7.9 ± 0.52^ns^	2.1 ± 0.14**	0.11 ± 0.078
RaPrP:Hep	NA	9.4 ± 0.61^ns^	0.24 ± 0.048**	0.12 ± 0.028
MuPrP:DS	4.8 ± 0.53*	0.66 ± 0.13	0.59 ± 0.047	0.57 ± 0.026
RaPrP:DS	2.7 ± 0.30*	zero	0.36 ± 0.091	0.46 ± 0.020
MuPrP:PA	0.054 ± 0.009^#^	0.30 ± 0.006	0.08 ± 0.003^&^	0.19 ± 0.01
RaPrP:PA	0.086 ± 0.003^#^	0.48 ± 0.048	0.18 ± 0.04^&^	0.13 ± 0.01
MuPrP:A1	0.17 ± 0.02	0.22 ± 0.03	0.17 ± 0.03	0.16 ± 0.02
RaPrP:A1	0.18 ± 0.03	0.29 ± 0.007	0.12 ± 0.01	0.13 ± 0.008
MuPrP:D67	0.28 ± 0.015	0.20 ± 0.02	0.21 ± 0.06	0.20 ± 0.05
RaPrP:D67	0.23 ± 0.08	0.41 ± 0.04	0.11 ± 0.009	0.11 ± 0.0007

We used linear regressions to fit fluorescence (F/F_0_) data from titration with GAGs and oligonucleotides. A one-site binding equation was used to fit fluorescence (ΔCM) data from titration with PA. The values shown are the mean ± SE from three experiments. Hep, heparin; DS, dermatan sulfate; PA, phosphatidic acid. A1 and D67 are oligonucleotides. NA, not analyzed; ns, not significant. *p = 0.0002, **p < 0.0001, ^#^*p* = 0.03, ^&^*p* = 0.001.

We did not observe any significant differences in terms of affinity when comparing the fluorescence data obtained for the interactions of MuPrP^23–231^ and RaPrP^23–231^ with heparin (Figure 5A) at pH 7.4. Meanwhile, we observed an eightfold affinity increase for MuPrP^90–231^ at pH 5.5 (Figure 5B and Table 1). Dermatan sulfate exhibited a low affinity for all PrP constructs (Figures 5C,D) except for PrP^23–231^ at pH 5.5 that it was twice as high for MuPrP than for RaPrP (Table 1). All GAGs had a greater affinity for PrP^23–231^ than PrP^90–231^.

### Evaluation of PA Interaction With Murine and Rabbit PrP

Lipids have also been identified in several studies as adjuvants of PrP conversion (Shyng et al., 1993; Nishina et al., 2004). To detect any differences in the interactions between RaPrP and a cofactor with different chemical and structural characteristics from GAGs, we also performed intrinsic fluorescence measurements to investigate the interaction with PA (Supplementary Figure 2). PA leads to an increase in fluorescence emission in all constructs at pH 7.4 and 5.5 (Supplementary Figure 2). This increase was accompanied by a blue shift of the emission spectra, indicating a shift to a more non-polar environment in the chemical environment of tryptophan. This suggests an interaction with the hydrophobic region of the LUVs and/or a reorganization of the structure where these amino acids are located (Supplementary Figure 2).

**FIGURE 6:**
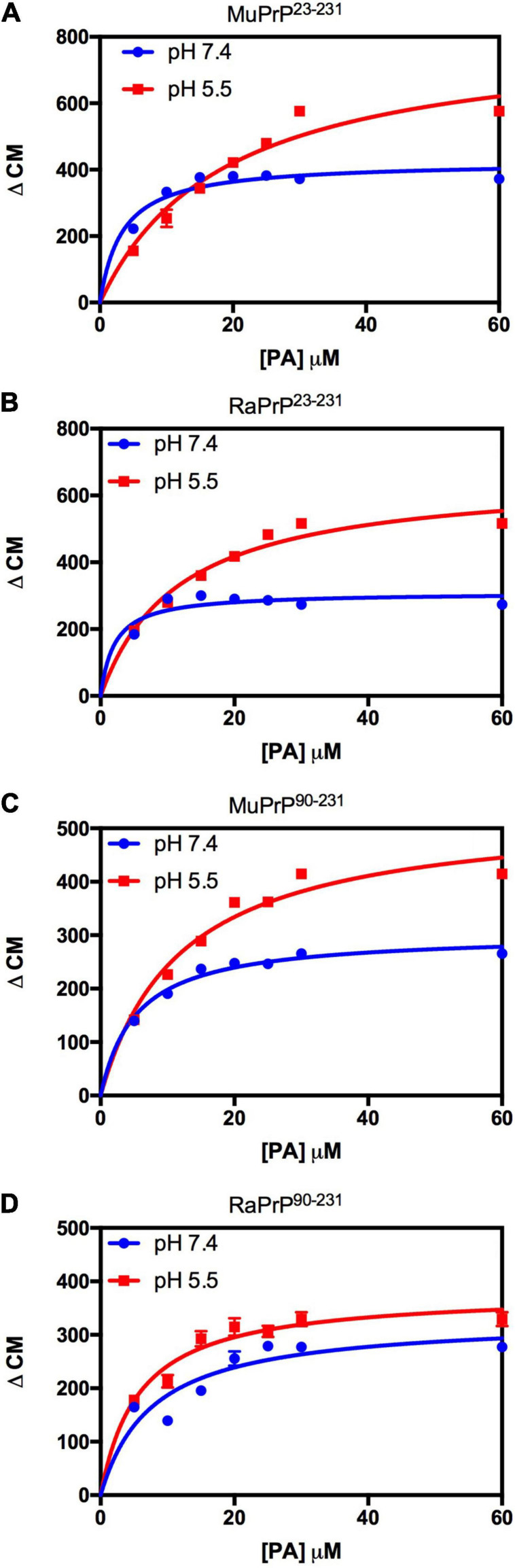
PA-induced structural change is enhanced at acid pH but to a lesser extent for rabbit PrP. Effect of increasing the concentration of PA on the center of mass values obtained from the fluorescence spectra of MuPrP^23–231^ **(A)**, RaPrP^23–231^ **(B)**, MuPrP^90–231^ **(C)**, and RaPrP^90–231^ **(D)** at pH 5.5 (red) and 7.4 (blue). All proteins were analyzed at 2 μM. The experiments were performed in 50 mM Tris buffer containing 100 mM NaCl at pH 7.4 or in 20 mM sodium acetate buffer containing 100 mM NaCl at pH 5.5. All experiments were performed at 25°C. The error bars represent the standard deviation of at least two independent experiments.

Variation of the center of mass (ΔCM) from intrinsic fluorescence data was obtained and fitted with a one-site binding non-linear curve, obtaining K_a_ values (Table 1) for all PrP constructs. Although ΔCM was greater at pH 5.5, the affinity was higher at pH 7.4 (Figures 6A–D and Table 1), suggesting that the structural changes observed as a result of the interaction do not depend directly on affinity. ΔCM and K_a_ were statistically different between pH values except concerning the interaction between PA and RaPrP^90–231^ (Figure 6C and Table 1). Affinity differences were only observed between MuPrP and RaPrP at pH 5.5, where RaPrP was 1.5-fold greater than MuPrP (Table 1). Affinity was also 1.5-fold greater in the presence of the complete N-terminal domain at pH 7.4 (Table 1). In PrP^90–231^, affinity is twice greater for RaPrP at pH 5.5 (Table 1).

**FIGURE 7:**
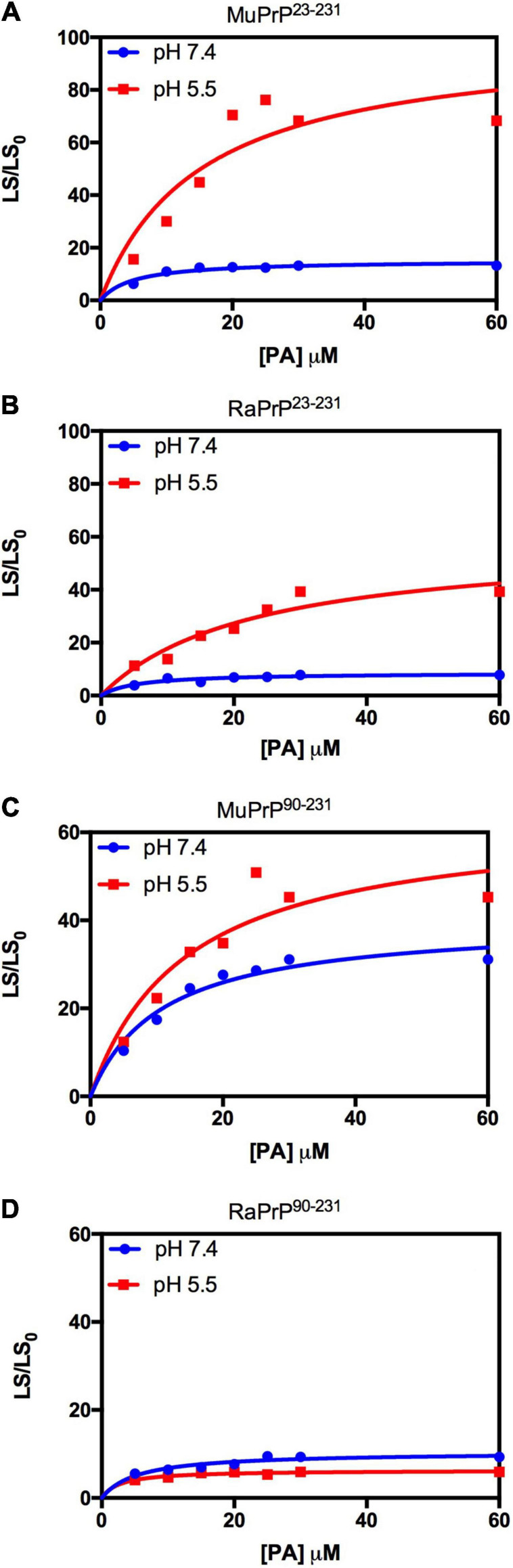
Rabbit PrP^90–231^ aggregates less in the presence of PA. Effect of increasing the concentration of PA on the relative light scattering of MuPrP^23–231^ **(A)**, RaPrP^23–231^ **(B)**, MuPrP^90–231^ **(C)**, and RaPrP^90–231^ **(D)** at pH 5.5 (red) and 7.4 (blue). All proteins were at 2 μM. The experiments were performed in 50 mM Tris buffer containing 100 mM NaCl at pH 7.4 or in 20 mM sodium acetate buffer containing 100 mM NaCl at pH 5.5. All experiments were performed at 25°C. The error bars represent the standard deviation of at least two independent experiments.

Light scattering measurements of MuPrP^23–231^ and RaPrP^23–231^ showed that PA was able to induce protein aggregation and that its effect was also pH-dependent (Figures 7A,B), with greater aggregation at pH 5.5. The aggregation of MuPrP (Figures 7A,C) was higher than RaPrP (Figures 7B,D) (175% for PrP^23–231^ and 900% for PrP^90–231^) at pH 5.5, despite the higher affinity observed for RaPrP (Table 1). This confirms that, in this case, structural changes and aggregation are not directly related to affinity.

### Interaction Between PrP C-Terminal Extended and DNA Oligonucleotides

DNA can bind with high affinity to the prion protein *in vitro* and modulate its aggregation (Cordeiro et al., 2001; Macedo et al., 2012). It has also been shown that the DNA structure leads to distinct interactions with MuPrP^90–231^ (Matos et al., 2020). In this study, we analyzed two DNA sequences previously described as high-affinity cofactors of MuPrP and evaluated whether they have different binding profiles in their interactions with MuPrP and RaPrP. We did this by monitoring intrinsic protein fluorescence and light scattering on a titration curve.

**FIGURE 8:**
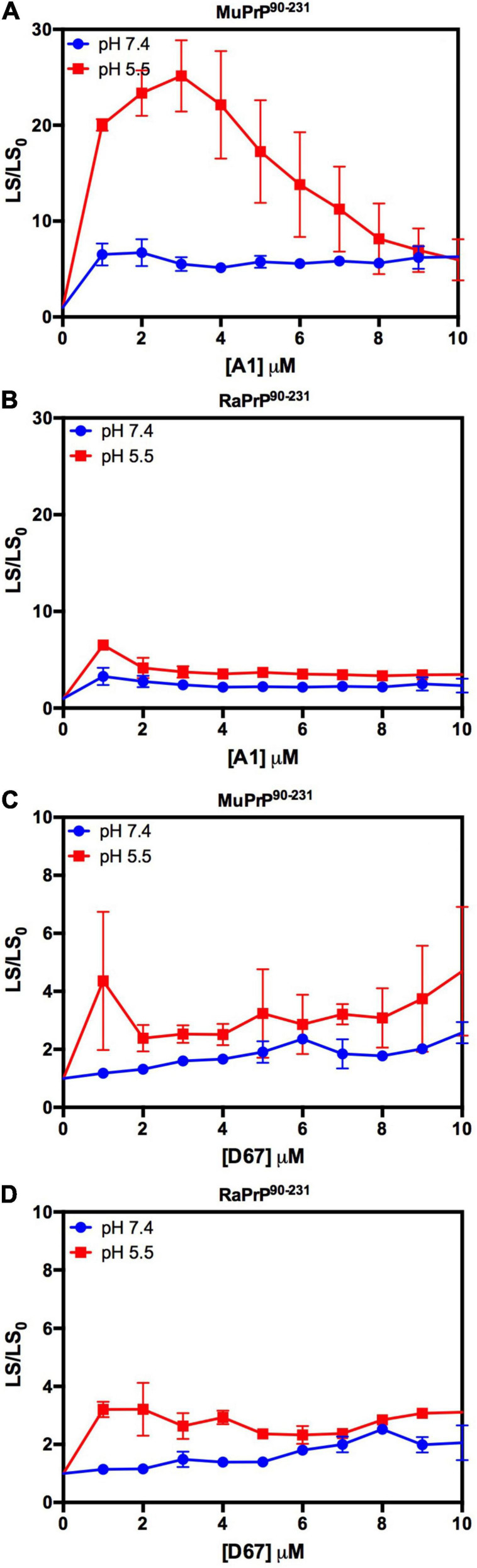
RaPrP^90–231^ aggregates less upon binding to DNA oligonucleotides. Effect of A1 **(A,B)** and D67 **(C,D)** on the relative light scattering of PrP^90–231^. Proteins used are MuPrP **(A,C)** and RaPrP **(B,D)** at pH 5.5 (red) and 7.4 (blue). The curves were performed with 5 μM PrP^90–231^ and increasing concentrations of oligonucleotides (from 1 to 10 μM). The experiments were performed in 50 mM Tris buffer containing 100 mM NaCl at pH 7.4 or 20 mM sodium acetate buffer containing 100 mM NaCl at pH 5.5. All experiments were performed at 25°C. The error bars represent the standard deviation of at least two independent experiments.

We began by evaluating the interaction of PrP^90–231^ and two DNA sequences previously studied by the group, A1 and D67, at two different pH values. There are evident differences when analyzing the light scattering of different combinations of the two proteins (MuPrP^90–231^ and RaPrP^90–231^) with both oligonucleotides (Figure 8). These changes are also linked to the pH, with acidic environments leading to four- to twenty-fold increase in light scattering for MuPrP^90–231^ and RaPrP^90–231^ when interacting with the DNA sequences. Aggregation was greater with A1 (Figures 8A,B) than with D67 (Figures 8C,D). A 20-fold increase was observed for the MuPrP^90–231^:A1 sample (Figure 8A) relative to the initial light scattering, while the increase in RaPrP^90–231^ was only sixfold (Figure 8B). The effect of D67 was less than A1 (Figures 8C,D). The same profile was observed at pH 7.4, though with a lower relative difference than at the lower pH.

**FIGURE 9:**
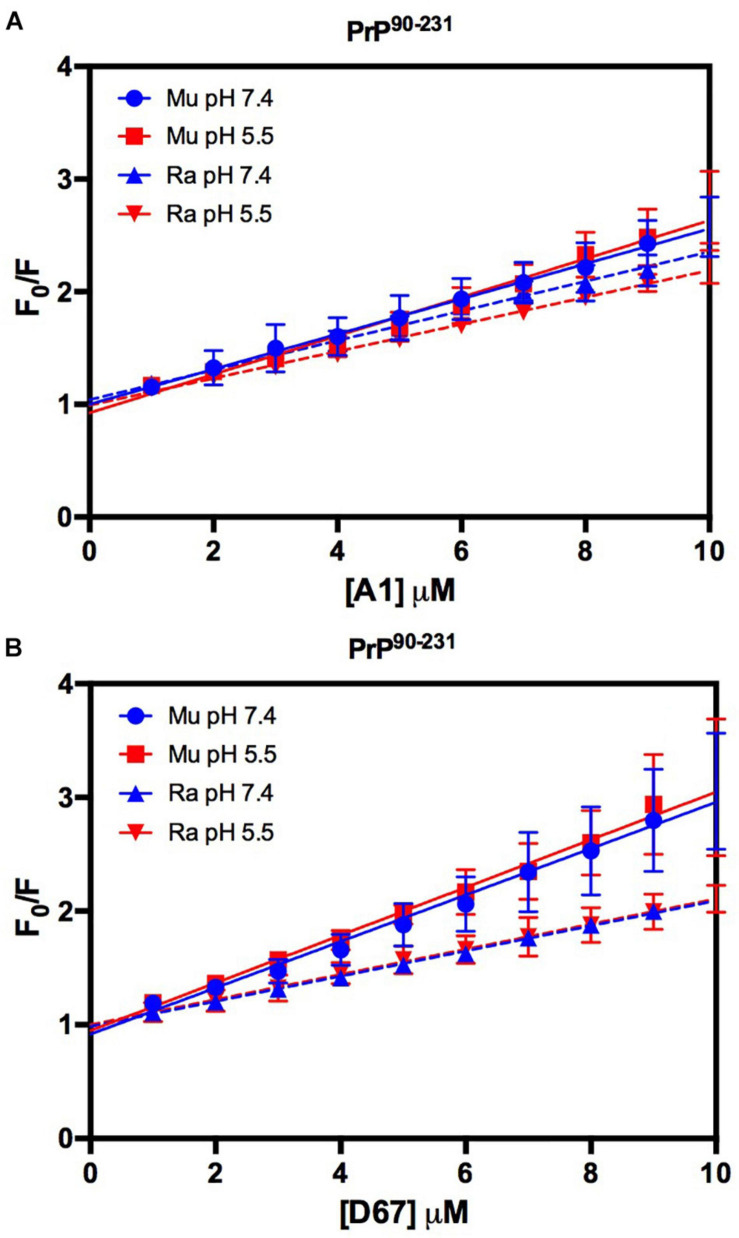
RaPrP^90–231^ interacts less with the DNA oligonucleotides. F_0_/F curves for titrations of A1 **(A)** and D67 **(B)** on PrP^90–231^ at pH 5.5 (red) or 7.4 (blue). The curves were performed with 5 μM PrP^90–231^ and increasing concentrations of oligonucleotides (from 1 to 10 μM). The experiments were performed in 50 mM Tris buffer containing 100 mM NaCl at pH 7.4 or 20 mM sodium acetate buffer containing 100 mM NaCl at pH 5.5. All experiments were performed at 25°C. The error bars represent the standard deviation of at least two independent experiments.

The intrinsic fluorescence quenching upon binding with oligonucleotides revealed that MuPrP^90–231^ had a higher affinity than RaPrP^90–231^ for both A1 (1.2 and 1.4 times at pH 7.4 and 5.5, respectively) and D67 (1.8 and 1.9 times at pH 7.4 and 5.5, respectively) (Figure 9 and Table 1). However, in contrast to the light scattering results, the differences between the samples containing A1 were not significant. The association constants show that the MuPrP:D67 interaction has the highest affinity of any treatment (Table 1).

To prove that any quenching effect observed was related to the addition of cofactors, the PrP^90–231^ fluorescence was evaluated with the addition of only reaction medium (Supplementary Figure 3). The fluorescence was not suppressed in a manner consistent with the observations for cofactors, GAGs or DNA.

### Interaction Between Full-Length PrP and DNA Oligonucleotides

In addition to the nucleic acid binding sites also present in PrP^90–231^, PrP^23–231^ has an additional predicted binding site in the lysine-rich region (residues 23–27) located at the extreme N-terminus (Yin et al., 2008; Cavaliere et al., 2013). We therefore sought to assess whether this region would change the interactions with the two DNA sequences.

**FIGURE 10:**
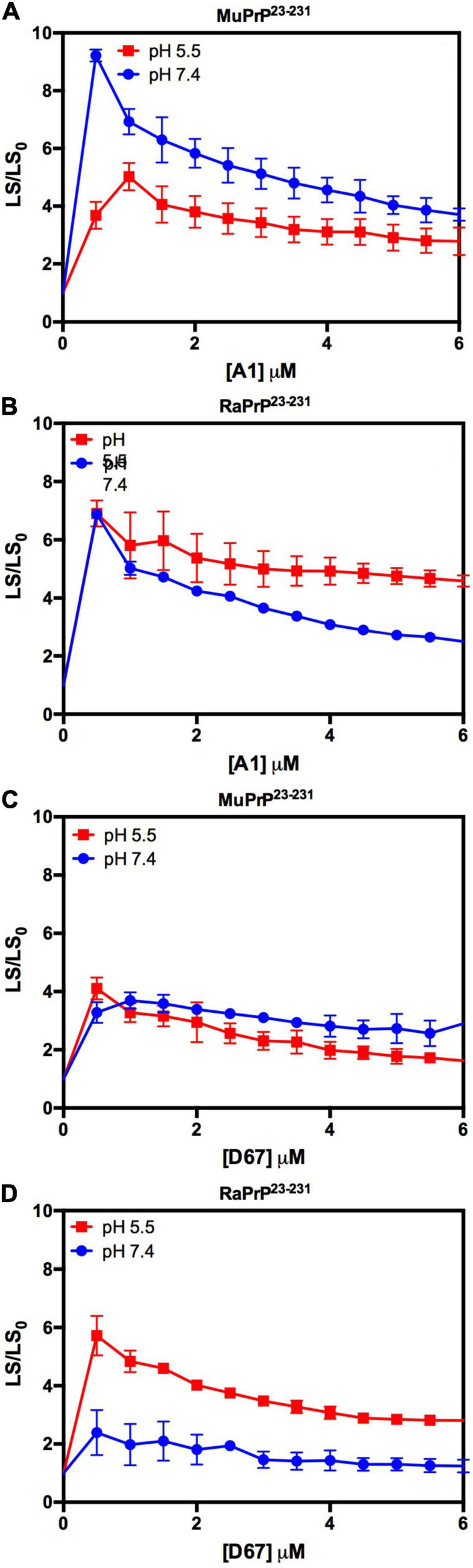
Interaction of PrP^23–231^ with DNA oligonucleotides causes an increase in light scattering. Effect of A1 **(A,B)** and D67 **(C,D)** on the relative light scattering of PrP^23–231^. Proteins used are MuPrP **(A,C)** and RaPrP **(B,D)** at pH 5.5 (red) and 7.4 (blue). The curves were performed with 2 μM PrP^23–231^ and increasing concentrations of oligonucleotides (from 0.5 to 6 μM). The experiments were performed in 50 mM Tris buffer containing 100 mM NaCl at pH 7.4 or 20 mM sodium acetate buffer containing 100 mM NaCl at pH 5.5. All experiments were performed at 25°C. The error bars represent the standard deviation of at least two independent experiments.

Depending on the aptamer used, we observed a 2- to 9-fold increase in the relative light scattering in the lowest stoichiometric ratio (Figure 10). In contrast to the results for PrP^90–231^, the light scattering of RaPrP^23–231^ with both oligonucleotides at pH 5.5 had a slightly higher scattering than the MuPrP^23–231^ samples (Figure 10). The profile for the two DNA sequences was similar to the observations for PrP^90–231^: A1 induced more aggregation than D67. Another difference between PrP^23–231^ and PrP^90–231^ was that the neutral pH treatments showed a higher relative light scattering for MuPrP (Figures 10A,C).

**FIGURE 11:**
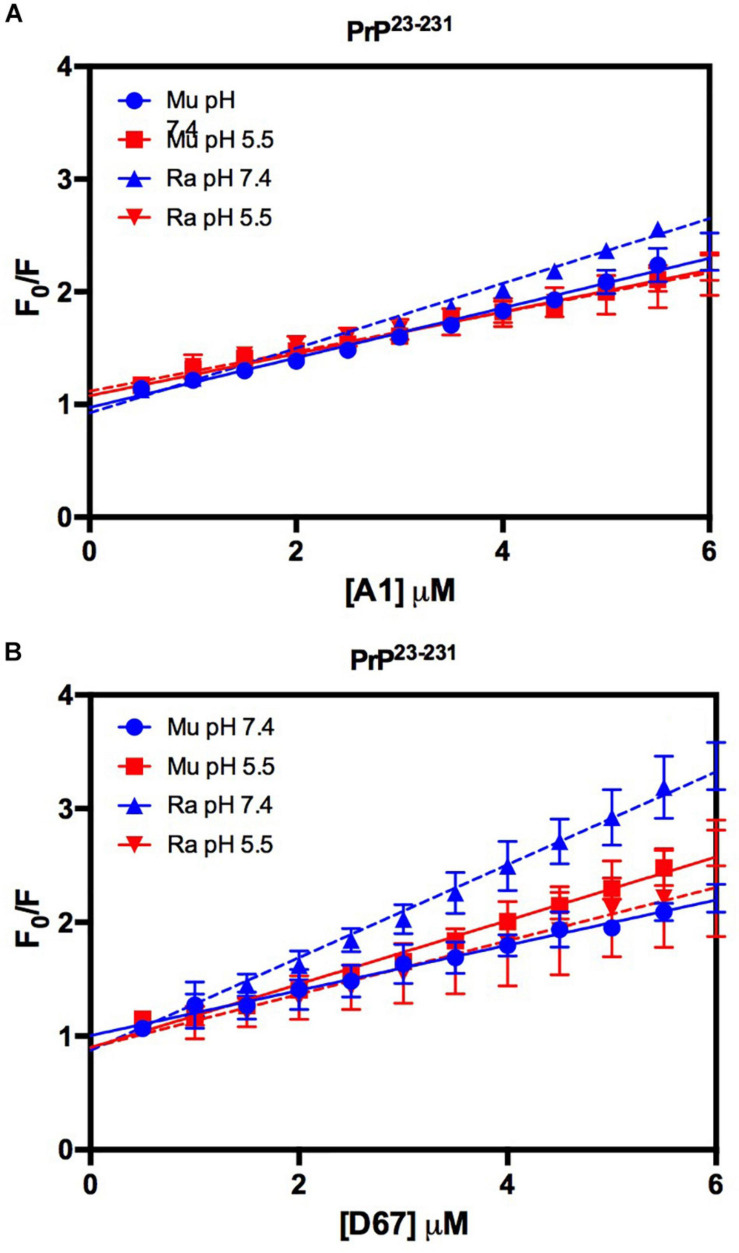
The intrinsic fluorescence of PrP^23–231^ is reduced upon binding to DNA oligonucleotides. F_0_/F curves for titrations of A1 **(A)** and D67 **(B)** on PrP^23–231^ at pH 5.5 or 7.4. The curves were performed with 2 μM PrP^23–231^ and increasing concentrations of oligonucleotides (from 0.5 to 6 μM). The experiments were performed in 50 mM Tris buffer containing 100 mM NaCl at pH 7.4 or 20 mM sodium acetate buffer containing 100 mM NaCl at pH 5.5. All experiments were performed at 25°C. The error bars represent the standard deviation of at least two independent experiments.

Similar to the findings for the PrP^90–231^ C-terminal extended protein, the interaction between DNA and the full-length PrP caused fluorescence suppression in every treatment analyzed (Figure 11). As expected, since PrP^23–231^ has more nucleic acid binding sites, the association constants were higher than those found for PrP^90–231^ (Table 1). Surprisingly, the interaction that showed the greatest association constant was between RaPrP and D67 at pH 7.4 (Table 1), which had the lowest light scattering increase (Figure 10D).

## Discussion

In this study, we compared the interaction of recombinant murine and rabbit PrP with different cofactors that have been identified as adjuvant molecules for conversion and aggregation of PrP, namely heparin, dermatan sulfate, PA, and DNA oligonucleotides (Tsiroulnikov et al., 2009; Silva et al., 2010). We show that RaPrP is less susceptible to aggregation by any of the cofactors tested, suggesting that the limited effect of these molecules may be associated with greater resistance to prion protein conversion observed in rabbits (Fernandez-Funez et al., 2011).

Recombinant rabbit PrP^c^ exhibits different electrostatic potential distribution than MuPrP, which has a larger positively charged surface (Wen et al., 2010a; Silva et al., 2011; Matos et al., 2020). This may affect interactions with negatively charged cofactors and, therefore, likely vary with pH. We found that all tested cofactors induce both MuPrP and RaPrP aggregation, and this effect does not correlate with differences on biding affinity, suggesting that the positive charge area found on RaPrP, covering residues 125–135, 150–160, and 180–190 (Wen et al., 2010a), is not related to RaPrP resistance to cofactor-induced aggregation.

The N-terminus of PrP does not seem to interfere with conversion to PrP^Sc^-like species, since truncated PrP can be converted, even if with lower efficiency (Lawson et al., 2001). However, this region is markedly different in rabbits (Figure 1; Myers et al., 2020), and is the main site for interaction with many of the studied cofactors (Silva et al., 2011). We observed the same effect for PrP^23–231^ and PrP^90–231^ for both MuPrP and RaPrP, although the overall interaction with cofactors was pronounced in the case of the full-length protein, suggesting that the N-terminal domain is determinant for interaction with cofactors resulting in an increased conversion efficiency, but not related to cofactor-induced aggregation resistance.

Horse PrP has four saline bridges (GLU196 -ARG156-HIS187, ARG156-ASP202, and GLU211-HIS177). RaPrP has a strong ASP177-ARG163 saline bridge, which keeps the β2-α2 loop attached (Zhang, 2011; Zhang and Zhang, 2014). ASP201-ARG155, which connects helices H3 and H1, makes two saline bridges in RaPrP, while MuPrP has only one (Figure 1). All these saline bridges contribute to the increased stability in RaPrP, especially at neutral pH, since losing these salt bridges at low pH reduces thermostability (Zhang, 2011; Zhang and Zhang, 2014). This effect of pH was consistent across the cofactors studied. However, even at low pH, aggregation of RaPrP was lower than that of the murine protein, suggesting that other interactions may be important.

Rabbit PrP SER173 forms a double hydrogen bond, forming a helix-capping motif, which decreases the tendency of RaPrP to populate a β-structured state at low pH (Khan et al., 2010). This may also contribute to RaPrP being less prone to cofactor-mediated aggregation, as observed here. The interaction site of low molecular weight heparin in the globular region of PrP is close to HIS186 (Vieira et al., 2011). Rabbit PrP shows a less dynamic hydrogen bond between HIS185 and ARG155 than murine PrP at neutral pH (Figure 1; Zhang, 2010, 2011). This HIS residue shows reduced pKa (∼5) in different species, and its protonation affects PrP stability. The HIS186ARG mutation, which introduces a positively charged residue, is correlated with familial CJD and destabilizes the murine protein (Singh and Udgaonkar, 2016). This more stable hydrogen bond may hinder the aggregation induced by low molecular weight heparin and other GAGs, although it does not prevent interaction due to residue protonation. The same must be important for the other studied cofactors. The introduction of a positive charge (due to protonation or residue mutation) should favor the interaction with negatively charged cofactors, such as those tested in this study, but the effect on RaPrP structural conversion and aggregation should be limited.

We observed some differences in the effects of the various cofactors tested. The aggregation profile of PrP in the presence of A1 and D67 was distinct from that in the presence of GAGs or PA. This may be due to differences in the regions of interaction, dissimilar charge and polarity, and/or even in the folding of these molecules. Intriguingly, the overall effect was identical.

We also evaluated the effect of a 21-mer double-stranded and of a 25-mer single-stranded DNA oligonucleotide (D67 and A1, respectively), on PrP aggregation. A1 induced higher aggregation than D67, possibly due to differences in binding affinity and/or DNA conformation (Macedo et al., 2012; M. Passos et al., 2021). Due to secondary structure, length, and sequence, nucleic acids may have different effects on PrP, in the extent of aggregation and toxicity of the aggregated species, as previously shown (Gomes et al., 2008; Macedo et al., 2012).

Interaction with PA induces PrP aggregation, but in a different manner than observed with GAGs and nucleic acids. The changes induced by PA led to a blue shift and increased fluorescence, suggesting more pronounced structural changes. PA interacted with full-length and truncated PrP, with a five-fold higher affinity at pH 7.4, contrasting with observations with phosphatidylserine, another anionic lipid, which actively interacted with human PrP^20–231^ at pH 5.0 but showed no interaction with human PrP^90–231^ (Morillas et al., 1999). The negative head group in PA may interact with positively charged amino acids. The N-terminal domain of PrP has two positively charged clusters, one between residues 23 and 30 and the other between residues 101 and 110, both of which are candidate sites for the interaction with PA, possibly explaining the differences observed with the two PrP constructs (full-length and truncated). Interestingly, dipolar phospholipids have also been shown to interact with and induce PrP aggregation (Kazlauskaite et al., 2003; Tsiroulnikov et al., 2009), indicating that the effect may not be necessarily related to the charge of the polar head group.

The present study is the first report of the direct interaction *in vitro* of dermatan sulfate with PrP. Dermatan sulfate is a GAGs found in the central nervous system, important for brain physiology, but is also involved in diseases that involve deficiency in the degradation of GAGs and consequent accumulation in endocytic vesicles - such as ataxia, intellectual disabilities, spasticity and other neurological symptoms are found in patients with mucopolysaccharidoses (Rauch and Kappler, 2006; Schwartz and Domowicz, 2018). Dermatan sulfate enhances the efficiency of PrP conversion by PMCA (Yokoyama et al., 2011), but there is no information available on its interaction with PrP and on the importance of this interaction for prion replication *in vivo*.

Our data also show that the interaction of PrP with dermatan sulfate leads to a similar effect to that observed with heparin, although with lower affinity and reduced aggregation. The interaction was not observed at pH 7.4, suggesting that structural differences between these GAGs are important at a neutral pH. Low molecular weight heparin binds to the octarepeat region of PrP at pH 7.4, and to helices H2 and H3 at pH 5.5 (Vieira et al., 2011). The fact that interactions with dermatan sulfate only took place at pH 5.5, with a higher affinity for PrP^23–231^, suggests that N- and C-terminal domains must be available to allow interaction. Interestingly, the differences observed for dermatan sulfate may be related to a relevant role in prion pathology.

## Conclusion

In summary, our findings demonstrate that most cofactors induce milder effects on RaPrP. Aggregation of RaPrP is weaker than that of MuPrP, specifically at acidic pH, suggesting that the mechanism involved in resistance to cofactors may be linked to RaPrP conformational/structural stability at low pH, and less to the physicochemical characteristics of the cofactors. The decreased effect of cofactors contributes to a better understanding of PrP conversion mechanisms and susceptibility among different mammalian species.

## Data Availability Statement

The original contributions presented in the study are included in the article/Supplementary Material, further inquiries can be directed to the corresponding author/s.

## Author Contributions

JA and YP: data collection and analysis and manuscript writing. JB: data collection. JS: data analysis and study design. YC: data analysis, study design, and manuscript writing. TV: data collection and analysis, study design, and manuscript writing. All authors read and approved the final manuscript.

## Conflict of Interest

The authors declare that the research was conducted in the absence of any commercial or financial relationships that could be construed as a potential conflict of interest.

## Supplementary Material

Supplementary Figure 1 Effect of Hep (1 μM) **(A)** and DS (1 μM) **(B)** on the aggregation of RaPrP^23–231^ (0.5 μM) at pH 5.5 monitored by light scattering (LS) over time. **(C)** Turbidimetry of disaggregated samples acquired on a spectrophotometer at 600 nm, after 18 h of incubation in low binding Eppendorfs. The experiments were performed in 50 mM Tris buffer containing 100 mM NaCl at pH 7.4 or 20 mM sodium acetate buffer containing 100 mM NaCl at pH 5.5. All experiments were performed at 25°C. Representative data from three experiments.

Supplementary Figure 2 Interaction with PA leads to increased tryptophan fluorescence and spectral shift to lower wavelengths. **(A–D)** Spectra based on the intrinsic tryptophan fluorescence emissions of murine **(A,C)** and rabbit **(B,D)** PrP^23–231^ **(A,B)** and PrP^90–231^ **(C,D)**. Self-normalized spectra of murine **(E,G)** and rabbit **(F,H)** PrP^23–231^ **(E,F)** and PrP^90–231^ **(G,H)**. Solid lines represent PrP alone. Dashed lines represent PrP in the presence of PA. All proteins were at 2 mM and PA at 30 mM. The experiments were performed in 50 mM Tris buffer containing 100 mM NaCl at pH 7.4 or in 20 mM sodium acetate buffer containing 100 mM NaCl at pH 5.5. All experiments were performed at 25°C. Representative data from three experiments.

Supplementary Figure 3 PrP^90–231^ intrinsic fluorescence reduction effect is caused by DNA oligonucleotides. Percentage curves of the PrP total intrinsic fluorescence intensity for titrations at pH 5.5 or 7.4. **(A)** MuPrP^90–231^ and **(B)** RaPrP^90–231^ 5 μM PrP^90–231^. The experiments were performed in 50 mM Tris buffer containing 100 mM NaCl at pH 7.4 or 20 mM sodium acetate buffer containing 100 mM NaCl at pH 5.5. All experiments were performed at 25°C.
