# Supplementary material for: Rabbit PrP Is Partially Resistant to in vitro Aggregation Induced by Different Biological Cofactors
Source: Front Neurosci. 2021 Jun 18;15:689315. doi: 10.3389/fnins.2021.689315 (PMC8249948; doi:10.3389/fnins.2021.689315)
Supplement: Supplementary Figure 1 — Effect of Hep (1 μM) (A) and DS (1 μM) (B) on the aggregation of RaPrP23–231 (0.5 μM) at pH 5.5 monitored by light scattering (LS) over time. (C) Turbidimetry of disaggregated samples acquired on a spectrophotometer at 600 nm, after 18 h of incubation in low binding Eppendorfs. The experiments were performed in 50 mM Tris buffer containing 100 mM NaCl at pH 7.4 or 20 mM sodium acetate buffer containing 100 mM NaCl at pH 5.5. All experiments were performed at 25°C. Representative data from three experiments. [file Image_1.pdf]

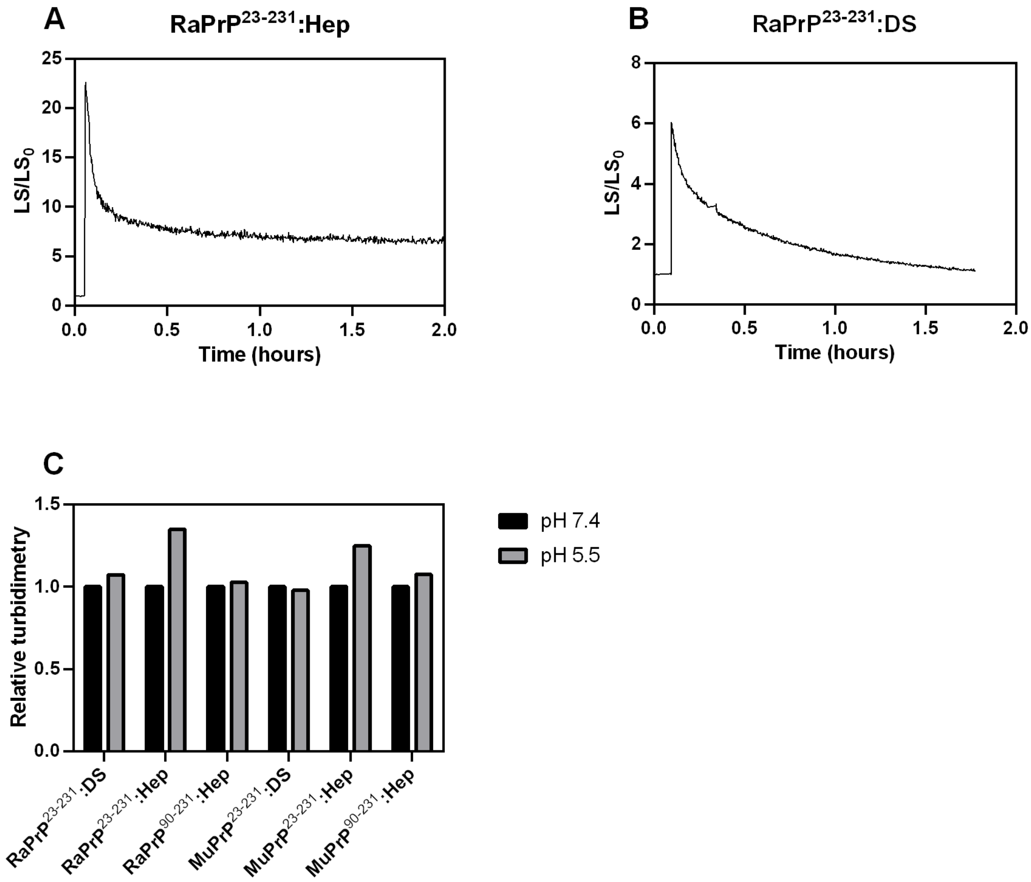

Supplemental Figure 1 - Effect of Hep (1 µM) (A) and DS (1 µM) (B) on the aggregation of $RaPrP^{23\text{-}231}$ (0.5 µM) at pH 5.5 monitored by light scattering (LS) over time. (C) Turbidimetry of disaggregated samples acquired on a spectrophotometer at 600 nm, after 18 hours of incubation in low binding Eppendorfs. The experiments were performed in 50 mM Tris buffer containing 100 mM NaCl at pH 7.4 or 20 mM sodium acetate buffer containing 100 mM NaCl at pH 5.5. All experiments were performed at 25°C. Representative data from three experiments.

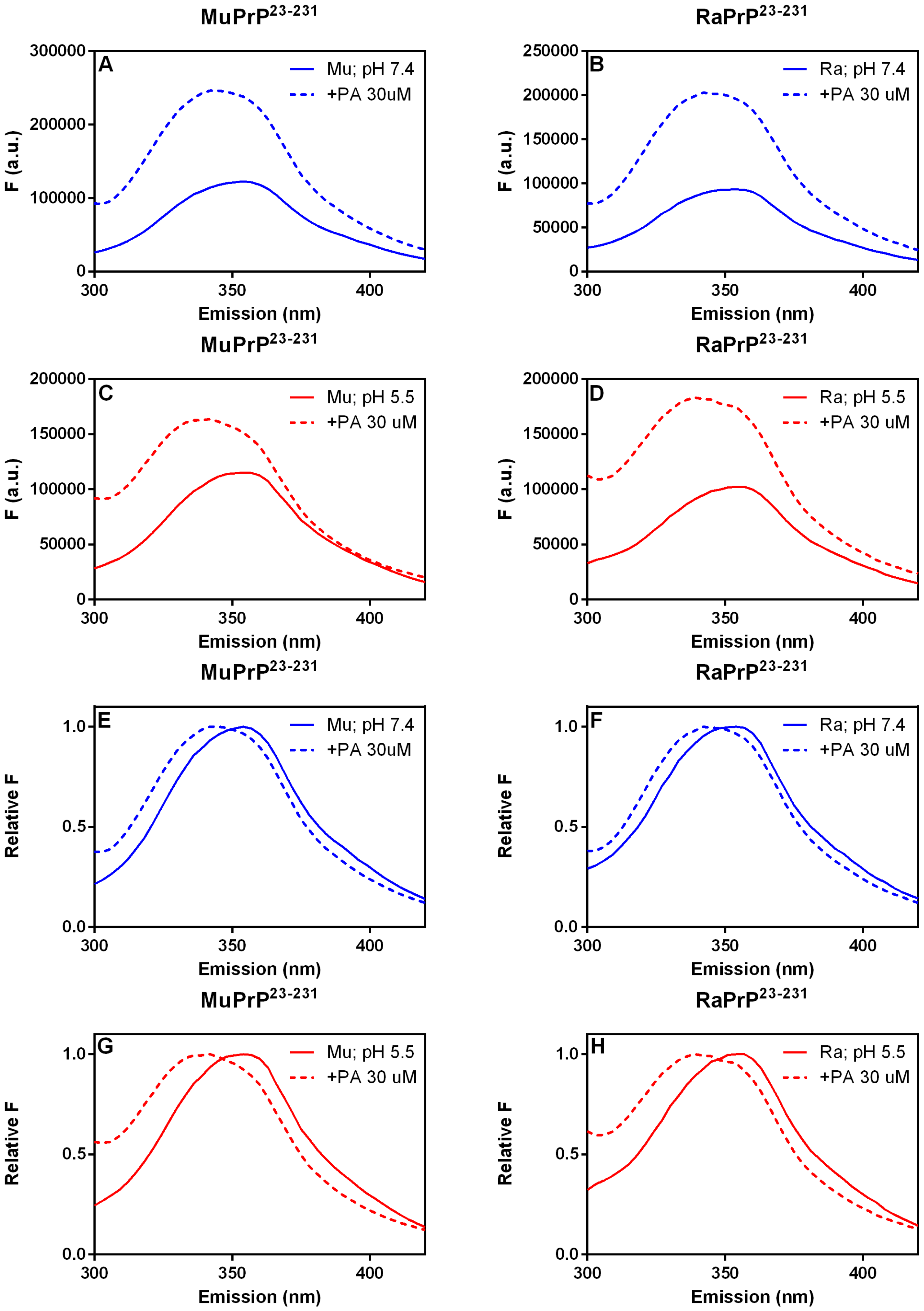

Supplementary Figure 2 – Interaction with PA leads to increase tryptophan fluorescence and spectral shift to lower wavelengths. A-D) Spectra based on the intrinsic tryptophan fluorescence emissions of murine (A and C) and rabbit (B and D) $PrP^{23\text{-}231}$ (A and B) and $PrP^{90\text{-}231}$ (C and D). Self-normalized spectra of murine (E and G) and rabbit (F and H) $PrP^{23\text{-}231}$ (E and F) and $PrP^{90\text{-}231}$ (G and H). Solid lines represent PrP alone. Dashed lines represent PrP in the presence of PA. All proteins were at 2 μM and PA at 30 μM. The experiments were performed in 50 mM Tris buffer containing 100 mM NaCl at pH 7.4 or in 20 mM sodium acetate buffer containing 100 mM NaCl at pH 5.5. All experiments were performed at 25°C. Representative data from three experiments.

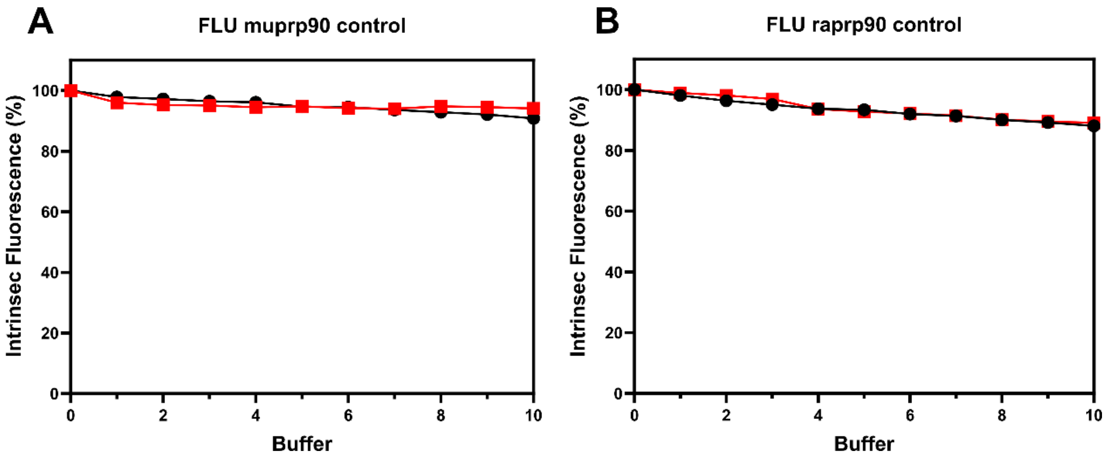

Supplementary Figure 3. PrP$^{90\text{-}231}$ intrinsic fluorescence reduction effect is caused by DNA oligonucleotides. Percentage curves of the PrP total intrinsic fluorescence intensity for titrations at pH 5.5 or 7.4. (**A**) MuPrP$^{90\text{-}231}$, (**B**) RaPrP$^{90\text{-}231}$ 5 µM PrP$^{90\text{-}231}$. The experiments were performed in 50 mM Tris buffer containing 100 mM NaCl at pH 7.4 or 20 mM sodium acetate buffer containing 100 mM NaCl at pH 5.5. All experiments were performed at 25°C.
